# The Circularity of the Embodied Mind

**DOI:** 10.3389/fpsyg.2020.01707

**Published:** 2020-08-12

**Authors:** Thomas Fuchs

**Affiliations:** Phenomenological Psychopathology and Psychotherapy, Psychiatric Clinic, Heidelberg University, Heidelberg, Germany

**Keywords:** embodiment, lived body, body–body problem, brain, circularity, circular causation, ecology, development

## Abstract

From an embodied and enactive point of view, the mind–body problem has been reformulated as the relation between the lived or subject body on the one hand and the physiological or object body on the other (“body–body problem”). The aim of the paper is to explore the concept of circularity as a means of explaining the relation between the phenomenology of lived experience and the dynamics of organism–environment interactions. This concept of circularity also seems suitable for connecting enactive accounts with ecological psychology. It will be developed in a threefold way:

(1) As the *circular structure of embodiment*, which manifests itself (a) in the homeostatic cycles between the brain and body and (b) in the sensorimotor cycles between the brain, body, and environment. This includes the interdependence of an organism’s dispositions of sense-making and the affordances of the environment.

(2) As the *circular causality*, which characterizes the relation between parts and whole within the living organism as well as within the organism–environment system.

(3) As the *circularity of process and structure* in development and learning. Here, it will be argued that subjective experience constitutes a process of sense-making that implies (neuro-)physiological processes so as to form modified neuronal structures, which in turn enable altered future interactions.

On this basis, embodied experience may ultimately be conceived as the integration of brain–body and body–environment interactions, which has a top-down, formative, or ordering effect on physiological processes. This will serve as an approach to a solution of the body–body problem.

## Introduction

According to enactive and ecological approaches to cognition, the mind is not to be regarded as a disembodied internal representation of the external world, nor as a system of brain modules, neural symbols, and algorithms that allow us to calculate and predict the world. On the contrary, an embodied mind manifests and integrates the current state of the entire organism as it interacts with its environment. Strictly speaking, it is not a “mind” at all, if by this is meant a separate domain or entity; it is rather a bodily subject whose experience extends over the lived body, and who, via its mediation, is in contact with the world (Thompson, 2007; Fuchs, 2018; Gallagher, 2018). In other words, the subject actually inhabits the body; I am co-extensive with my body, and its movements are literally my movements – not some external events for which the brain simply creates a suitable body phantom that I happen to experience. The body is not a mere vehicle but the very locus of the subject, the source, and the medium of its relation to the world.

If we thus re-conceptualize the disembodied mind, which is still the predominant concept of present-day Cartesian materialism (Rockwell, 2005; Knowles, 2014), then the mind–body problem has to be recast. It is no longer a question of how the mind is related to the brain but how the lived or subject body on the one hand is related to the living or object body on the other; in short, it becomes the “body–body problem,” as Hanna and Thompson (2003) and Thompson (2007) have termed it. A particularly challenging aspect of this problem is the question of whether and how we may attribute a more than epiphenomenal role to bodily subjectivity.

In what follows, I want to address this problem from several points of view. First, I will present the ontological relation between lived body and living body in terms of a *dual aspect* of the living being. Then I will use the concept of *circularity* to describe the relation and intertwinement of both aspects. As I will try to show,

(1) Circularity characterizes the structure and dynamics of the living organism on different levels, thus giving rise to the lived body;
(2) Circular causality, or downward and upward causation, characterizes the part–whole relation of the organism, enabling the actual effectiveness of embodied subjectivity in the world; and
(3) The circularity of process and structure shapes the development of the living being over time. This will lead finally to a proposal as to how, in humans, this development may be increasingly determined by the embodied subject itself.

## Lived Body and Living Body

My starting point is the circular relation between lived body and living body, or subject body (*Leib*) and object body (*Körper*). The lived body is mostly transparent to us: it is the pre-reflective background and medium of our world-directed perspective, the center from which we see, act, and live without paying attention to it. The object body appears in our experience when this perspective is turned backwards; this happens with all conspicuous bodily sensations, but in particular when fluent bodily functioning is disturbed or interrupted, be it through a mishap, clumsiness, exhaustion, or illness. In such cases, the body is no longer transparently lived as mediating our activity in the world. It becomes “an explicit part of the subject’s experiential world rather than its implicit mode of revealing that world” (Stapleton and Froese, 2016, p. 124).

On the other hand, the living or object body (now regarded from a third-person perspective) constitutes the subject body, inasmuch as the organic functions tacitly enable the latter’s mediating role for our activities. Thus, living and lived body are in a relation of mutual concealment, because they bring forth or constitute each other, and this is what defines our embodiment. A well-known manifestation of this reciprocal relation is the phenomenon of double touch as highlighted by Husserl (1952): if one’s right hand touches the left, the latter appears as a *palpable object* offering resistance to the right hand’s touch (i.e., as *Körper*); however, through a change of attention, it can also become a *feeling hand*, sensing the touch, that is, a part of the bodily subject (*Leib*).

**FIGURE 1:**
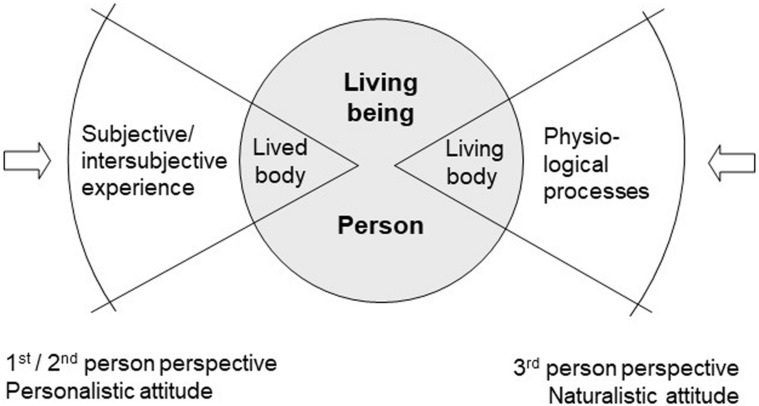
Dual aspect of the living being (adapted from Fuchs, 2011).

This example shows that lived body and living body correspond to two different *perspectives or attitudes* between which we shift in everyday life, usually without being aware of it. Nevertheless, both perspectives are related to one and the same living being, a living being that displays two different *aspects.* This fundamentally changes the usual construal of the mind–body problem: it is generally based on the principal divide between a “mental” sphere and a “physical” sphere, the one being only accessible from within, or from the first-person perspective, the other only accessible from without, or from a third-person perspective. Instead of such a gap between two radically different ontologies (the mental and the physical), we are now faced with a *duality of aspects within embodiment* (Fuchs, 2018, pp. 77–82). The question, then, is about the relation between one’s body as a living organism and one’s body as subjectively lived. And the answer must be that processes of *living* and processes of *experiencing* (in German: *Leben* and *Erleben*) are both aspects of the organism’s life process seen from different but complementary points of view. On this understanding, the living being or animal becomes the ontological basis for embodied subjectivity on the one hand and for the objective body considered by physiology on the other. They are both complementary yet irreducible and mutually concealing aspects of the living being, like two sides of a coin (Figure 1).

A first consequence of this is that in order to grasp the embodied mind, we have to extend the narrow focus taken by neuroscience on the brain and take instead a wider view. *Only the living being as a whole* may be regarded as the proper subject of feeling, thinking, speaking, acting, and so forth. Neuronal activations or circumscribed brain structures are not the adequate scale at which to look for the basis of the mind. Rather, it is only through interacting with others in an empathic mode, or from a second-person perspective, that we get access to the embodied mind of the other. Narrowing the focus and getting ever closer to the physical body and its component parts mean a shift from what Husserl (1952) called the “personalistic” to “naturalistic attitude” or from the second- to third-person perspective (Figure 1). From this perspective, however, embodied subjectivity no longer shows itself.

On a daily basis, a doctor undertakes this change in attitudes, for instance, when greeting a patient and seeing his (friendly, anxious, or similar) gaze, yet shortly afterward taking hold of the ophthalmoscope to examine the patient’s eyes as physical organs: at this point, looking at them from too close a distance, the gaze has vanished. The embodied subject is only perceivable as a whole. The doctor may get still closer and investigate the retina – just like a physiologist or a neuroscientist may explore all the microstructures and microprocesses of the physical body, for example, the visual cortex. Yet nowhere will consciousness, mind, or life show themselves – they are *macro-phenomena*, which are only accessible to others in coexistence, from the second-person perspective^1^.

Nevertheless, both attitudes are directed to the same entity, that is, the living being or the living person. The lived or subjective body as the location of sensations and affections (fatigue, pain, hunger, etc.), as the medium of the enactment of life or of contact with others – none of this emerges as a construct in the brain, mysteriously projected into external space. Rather, this lived body *is the organism itself* under the aspect of a holistic aliveness that is manifested both subjectively and intersubjectively. We can thus consider the same entity in much the same way as a reversible figure such as Necker’s Cube, in two distinct and non-transferable ways – as the lived body and as the physical body.

In sum, taking an embodied and enactive approach implies extending one’s view, both with regard to space and time: looking at the wider system and how it develops over time. Then we can see both experiential and physiological processes, the lived body and the physical body as belonging to a more encompassing system, namely, the system of the living being and its environment, or of the person and her world – an ecological system that is in continuous development (Lewin, 1951; Gibson, 1979).

## Circularity

I have presented a dual aspect concept of the living being, or more specifically of the human person, comprising the subjective body and the physical body. In order to further elucidate the relation between and intertwinement of both aspects, thus tackling the body–body problem, I will use the concept of *circularity*. As a first step, I will show that circularity characterizes the structure and dynamics of the organism on different levels, thus giving rise to the subjective body. In a next step, circular causality will be seen to help explain the actual significance and effectiveness of the subjective body for the self-sustainment of the living being.

### Interactive Cycles of the Embodied Mind

To begin with, there are two interactive or feedback cycles that form the basis of the embodied mind (Thompson and Varela, 2001):

(a) Cycles of organismic self-regulation, engendering a basic bodily sense of self; and
(b) Cycles of sensorimotor coupling between organism and environment, implying an “ecological self.”

Importantly, a concept of biological embodiment implies that the sensorimotor interaction (b) is deeply rooted in the organism’s internal self-regulation (a), or in phenomenological terms, the subject’s “being toward the world” (ecological self) is grounded on its bodily self-awareness (basic self). Thus, the living body is not just a mechanical device of sensorimotor input and output; otherwise, it would not be distinct from a robot body as conceived in embodied AI (Ziemke, 2016). The body is rather animate, it feels and senses itself, and this self-affection is the basis of its perceiving and acting relation to the environment. This will become clearer in the following.

#### Cycles of Organismic Self-Regulation

As is well-known, the self-sustainment of the organism depends on homeodynamic regulatory cycles involving the brain and body at multiple levels. However, organismic regulation also has a dimension of basic self-affection or self-awareness. Affective neuroscience, represented by authors like Damasio (1995, 1999, 2010) and Panksepp (1998, 2005), has emphasized the dependence of a *background consciousness* on the homeodynamic regulation of the entire body: various centers in the brain stem, hypothalamus, and insular and medial parietal cortices process the proprioceptive, visceral, vasomotor, endocrine, and other afferences from the internal body and integrate them into a “body landscape” that is constantly changing. This landscape includes the present state of the inner milieu (e.g., heart rate, blood pressure, blood oxygen, glucose, temperature, intestinal movements, vestibular sensations, and muscle tension). In this way, the inner milieu is continuously registered as *interoception* (Craig, 2002, 2003). Conversely, the organism’s homeostasis is constantly regulated by the brain via descending innervations (parasympathetic and sympathetic nervous system) as well as via hormone secretions from the hypothalamus and the pituitary. This results in what may be called an “interoceptive loop.”

The brain and body are therefore most intimately connected and influence each other in constant circular feedback. This interaction brings forth an interoceptive *feeling of being alive* (Damasio, 1995, p. 150): a basic self-affection with the hue of comfort or discomfort, pleasure of displeasure, relaxation or tension, or other basic moods. The feeling of being alive corresponds to a basic bodily self-affection or *a minimal form of subjectivity* (Fuchs, 2012a). Processes of life and processes of mind are thus inseparably linked: all conscious states are ultimately rooted in the homeodynamic regulation between the brain and body and, in a sense, integrate the present state of the organism as a whole. The foundation of subjectivity thus lies in the visceral or “deep body” and its vital self-regulation (see also de Preester, 2007). This may be considered as an organismic basis for the life–mind continuity thesis supported by enactivism (Thompson, 2007; Froese and Di Paolo, 2009; Kirchhoff and Froese, 2017).

A frequent objection to such an account refers to a representational and internalist concept, according to which the state of the body is mapped or modeled in the brain, thus only serving as external input. This is indeed Damasio’s position as well, for example, when he claims that the basic or protoself is constituted by “mental images of the body produced in body-mapping structures” in the brain (Damasio, 2010, p. 21). This would mean that self-awareness and consciousness are ultimately located within the brain. On the other hand, Damasio himself speaks of a continuous “resonant loop” between the brain and body (Damasio, 2010), which is hardly reconcilable with a representationalist account in the traditional sense, because “resonance” is obviously different from “internal modeling.” Elsewhere, Damasio also describes the process as

“(…) a looped circuit where the body communicates to the central nervous system and the latter responds to the body’s messages. *The signals are not separable from the organism states where they originate.* The ensemble constitutes a dynamic, bonded unit (…) this unit enacts *a functional fusion of body states and perceptual states*, such that the dividing line between the two can no longer be drawn (…) the signals conveyed would not be *about* the state of the flesh but literally *extensions of the flesh*” (Damasio, 2010, p. 273; my italics).

Within such a looped circuit or functional fusion, however, there is *neither place nor time for a separate representation*. There is no component within the circuit that represents another one, in the sense *that it could stand for it while it is absent* (“the signals … would not be *about* the state of the flesh”). The term representation suggests that the brain activities could in principle be separated from the circuit, as if they were reconstructing inside the brain what is outside^2^. But in the functional fusion of the body and brain described by Damasio, there is no longer any inside and outside. Hence, Damasio’s representationalist account seems self-contradictory, and instead of a representative or mapping relation, we should rather speak of a *continuous mutual resonance* between the brain and body. If that is the case, then primary self-awareness can no longer be localized anywhere in the brain; rather, it is the integral manifestation of the *brain–body system*, or of the overarching process of life, which encompasses the whole organism^3^. The same applies to emotions: as resonant loops between the brain, body, and environment, they are no longer the brain’s representations of the body’s activity, as Damasio puts it, but rather *the feelings of the body itself* vis-à-vis a certain situation (on a corresponding circular model of embodied affectivity, see Fuchs and Koch, 2014; Fuchs, 2018, pp. 120–125).

#### Sensorimotor Cycles

Embodied subjectivity does not stop at the boundaries of the skin but is extended as “being toward the world” (Merleau-Ponty, 1962), mediated by the habitual functioning or the “operative intentionality” of the body. In enactive terms, this corresponds to the structural coupling of organism and environment, produced by *functional cycles of sensorimotor interaction*. Here, the lived body is pre-reflectively experienced as the point of convergence of action and perception. Interoception is the basis of exteroception; the self-affection of the deep body provides the sense of mineness, which pervades all interactions with the world^4^. In this way, basic bodily self-awareness becomes a world-directed, extended consciousness.

As is well-known, the enactive approach to cognition regards perception as a process of active *sense-making*: by interacting with the environment (moving their head and eyes, touching a surface, walking toward a goal, grasping a fruit, etc.), living beings make sense of their surroundings (Varela et al., 1991; Thompson, 2005, 2007; Di Paolo et al., 2017). Sense-making has a circular structure: perception makes use of sensorimotor contingencies (O’Regan and Noë, 2001; Noë, 2004), namely, by skillfully exploring the environment (looking, touching, etc.) and then grasping the results. For this circular intertwinement of perception and action to work, the body’s own movement has to be *self-referential* or *self-given* through kinesthesia and through “efference copy” mechanisms^5^.

These interconnections of perception and movement include a *temporal circularity* as well. In phenomenological terms, each bodily action implies anticipations or *protentions* (being prepared for the response of the environment) that may or may be not fulfilled in subsequent perceptions (Behnke, 2009). Thus, protention and response form a temporal circle that extends into the future. Similarly, objects are always perceived as enabling possible actions, or in Heidegger’s terms, as objects “ready to hand” (Heidegger, 1962). This is captured, in ecological psychology, by Gibson’s term *affordances* (Gibson, 1979), which are objective structures of usefulness or viability provided by the environment. “The uses of things are directly perceived” (Gibson, 1982, p. 409), but this perception is at the same time a perception of *future possibilities* that correspond to the body’s capacities and protentions. An object such as a knife can only be perceived by an embodied agent capable of somehow interacting with it, for example, by having suitable limbs to walk toward the knife, grasp it, and so forth, thus perceiving the knife as an affordance structure. In a way, the knife is a unity of present and future. Indeed the entire body (and by no means only the brain) may be regarded as a *system of expectations and “predictions*,*”* which make sense of the environment as a space of potentialities or affordances and their possible fulfillment^6^.

This anticipatory structure may be considered as an extension of the organismic self-regulation at the level of the deep body. Homeostasis is now achieved not just by simple set point regulation but also through external sensorimotor loops by which the organism actively establishes and ensures the conditions of its self-sustainment. The circular structure of internal self-regulation is thus extended spatially as well as temporally: through anticipating possible satisfaction or danger, living beings are able to seek preferable situations and to avoid precarious ones – a crucial mark of their adaptivity (Di Paolo, 2009). As this goes beyond internal homeostasis, Sterling (2012) and Vernon et al. (2015) have introduced the suitable model of *allostasis* to describe a mode of self-regulation by anticipating needs and preparing to satisfy them *before* they arise. Allostasis is related to the future as a realm of possibilities and values. For these extended loops, drives and emotions play a crucial role: distant goals require a striving (or aversive) anticipation. “The animal has to span a gap that represents in time what the gap between itself and the relevant objects represents in space. The latter gap is provisionally spanned by perception, the former is by emotion” (Jonas, 2001, p. 104). Thus, hunting is motivated by appetite, desire, and aggression, whereas flight is driven by fear. Through emotions, affordances are perceived as *valuable*, for instance, as attractive or as repulsive.

#### Circularity of Affordances

The account of sense-making given so far also allows us to see affordances as having a dual aspect, as Gibson has suggested:

“[A]n affordance is neither an objective property nor a subjective property; or it is both if you like” (Gibson, 1979, p. 129).

The concept of circularity can be applied to this dual aspect of affordances, which are neither purely physical properties nor subjective mental projections:

– On the one hand, the living being makes sense of the environment as affording certain possibilities of action, namely, on the basis of needs and desires of the *lived body*; this is the subjective aspect of affordances.
– On the other hand, the environment objectively offers precisely these possibilities of interaction, thus providing a suitable “niche of affordances” for the *living* or *object body*. In the course of a concrete action, these affordances and their sensory flow continually define the body’s further sense-making activity (Fultot et al., 2016).

In other words, there is a circular interrelation between the needs of animals and the corresponding affordances in the environment, which are disclosed by these needs. This relation itself is an objective feature of the ecological system. Affordances are real, regardless of whether they are currently perceived or used. Thus, the structural coupling of organism and environment renders affordances *objective relational properties* in the world (see also Chemero, 2003). The dual aspect of lived body and living body allows us to consider these relations from both complementary perspectives.

#### The Role of the Brain

I have spelled out the animal’s sense-making in terms of spatial and temporal loops extending into the environment. It is obvious that these loops are not produced by the brain alone; they are crucially mediated by the whole body and its protentions. The brain functions rather as an *organ of suitable dispositions*: Through its networks, it provides *open loops* of possibility that are closed by suitable complements in the environment and thus become functional cycles of interaction (Fuchs, 2011, 2018). For example, there are so-called canonical neurons in the premotor cortex that are activated both when handling tools and when only looking at them (Grafton et al., 1997; Gallese and Umiltà, 2002). This means that the knife is perceived as “ready to hand” in an embodied sense, because the motor system and the hand are already involved in its perception as open loops. The same is demonstrated by handled objects priming the according reach and grasp actions (Masson et al., 2011).

However, the anticipatory structure of the action–perception cycle involves the *entire body* interacting with its environment and may not be reduced to a “predictive brain.” Open loops are neither “hypotheses” nor “predictions” about the world but rather dispositions of neural and bodily activity (shaped in the course of earlier sensorimotor experiences) that mediate the skillful coping with situations and objects. As long as their anticipatory structure is fulfilled, the functional cycles run smoothly (usually without conscious attention); if there is a mismatch, then an irritation or surprise results, now requiring conscious reorientation and adaptation. Therefore, neural processes should be described neither as internal representations nor as models or predictions but rather as dispositional patterns that participate in dynamic sensorimotor cycles involving the whole organism–environment system. The cycles run through the brain, body, and environment, leaving no separate “inside” and “outside” for representations to work. A more adequate concept would be based on the notion of *resonance* between the brain, body, and environment^7^.

Hence, if I skillfully handle a knife to carve a piece of wood, there is no boundary in the action that would separate the brain from my body, nor my body from the environment. Neural networks; muscular movements of my hand, knife, and wood synergically work together; and the whole resonating brain–body–environment system creates my experience of agency. Being able to carve is obviously a capacity not of the brain but of an embodied subject coupled to an environment that provides the necessary complements. This corresponds to the subjective experience of embodying the knife or any other tool into one’s body schema: I am not a pure consciousness outside of my own action but an embodied and “ecological self” whose borders do not stop at my skin (Neisser, 1988). Hence, consciousness may not be localized in any one place; it is the “integral” of the ongoing interaction and resonance between the brain, body, and environment^8^.

As we can see, from an enactive approach, *the phenomenology of bodily being in the world corresponds to the ecology of the organism in relation to its environment*. Lived body and physical body are both complementary aspects of the same life process that connects the living subject and the world, or the brain, body, and environment in circular interactions^9^.

### Circular Causality of Living Systems

As shown above, the basic self-awareness arising from the deep body forms the core of the body-as-subject. This core is extended as bodily “being toward the world,” where the body functions as medium of our sensorimotor interactions with the environment. Both the basic bodily self-awareness and the extended lived body may be regarded as the *integral* of the brain–body and the brain–body–environment cycles, respectively. The next question is whether these higher-level phenomena of bodily subjectivity also have an effectiveness of their own, or whether they are only epiphenomena of processes on the microlevel. Does the bodily experience of hunger or anxiety actually lead to the actions required to satisfy the hunger or avoid the anticipated threats?

A concept that is suitable for establishing the significance of the lived body is known as *circular causality*, also termed downward/upward causation or global-to-local/local-to-global causality (Haken, 1993; Thompson, 2007; Murphy et al., 2009; Vernon et al., 2015). Circular causality obtains between higher- and lower-level processes, or between the whole and the components of a system. Thus, a living being may be regarded as a system that continuously reproduces the components of which it consists (organs, cells, biomolecules, etc.), whereas these components reciprocally sustain and regenerate the system as a whole. The whole is the condition of its parts but is in turn realized by them.

Such a structure, for instance, characterizes the relations between genes and the organism: the genetic structure of an individual cell nucleus controls the necessary production of specialized cellular organs and functions (=upward or local-to-global causation). Conversely, the entire configuration and function of the organism are involved in defining which genes of the individual cell attain any relevance at all for its development, specialization, and regulation (=downward or global-to-local causation). Another example is as follows: an emotional state such as a patient’s anxiety can be treated pharmacologically, that is, by directly influencing the transmitter metabolism in the brain (upward). On the other hand, this can also be achieved by calming talk, that is, on the higher level of social interaction, which changes the patient’s perception of his or her situation (downward). As such, intersubjectivity corresponds to an integral level of organism–environment interactions that feeds back into lower-level (neuro-)physiological processes.

This type of causality is often criticized and rejected, on the grounds that it either presupposes unknown physical forces, thus contradicting the laws of physics, or that it is superfluous and falls prey to Occam’s razor (Craver and Bechtel, 2007). However, by no means are we obliged to restrict the notion of causality to effective causes (*causa efficiens*) as in the model of billiard balls acting on each other. Macrostructures may well develop formative or organizing effects with regard to the microelements in which they are realized, in accordance with Aristotle’s *causa formalis* (Juarrero, 1999, pp. 125–128). This does not mean that new forces emerge that would contradict physical laws. Rather, macrostructures are in a position, thanks to their form and configuration, to *select* specific properties and behaviors of their components and *block* others (Campbell, 1974; Moreno and Umerez, 2000).

Thus, these components acquire *emergent* properties, for instance, iron incorporated in hemoglobin. Normally, iron exposed to oxygen and humidity rusts, as it binds oxygen irreversibly. The process of respiration, however, crucially requires that the iron is in a position to incorporate oxygen reversibly, which would never happen in inorganic nature. This purpose is served by hemoglobin, a macromolecule consisting of about 10,000 atoms, with the sole purpose of enabling iron to release its oxygen in the necessary areas of the organism. For this to occur, no physical “miracle” is required, but only a superordinate organizational structure (in this case hemoglobin) that selects and “enslaves” its own compositional elements (Haken, 1993; Kelso, 1995), that is, integrates them into specific behavioral patterns. Generally, the molecular processes within a living cell are so constructed that they produce chemical reactions and molecules, which defy the odds of natural occurrence by many orders of magnitude (Deacon, 2006). Thus, the form, configuration, or topology of a living system constrain the *range of possibilities* in the system’s phase space.

Analogously, mental processes, as embodied and integral acts of a living organism, can be effective in that organism’s physical behavior. Of course, subjectivity does not affect physiological processes as an external force but rather exerts a top-down formative influence over them. If I, for instance, speak a sentence, the muscles of my tongue and larynx display organized patterns of movement. Their proximate or efficient cause is the release of acetylcholine at the motor endplates of these muscles. Nevertheless, it is equally correct to say that my tongue and larynx move in these ways *because I am speaking these words* and I am intentionally directed toward their content. This “because,” however, no longer signifies an efficient, but a higher-order *selecting and forming cause:* the muscles are always ready for excitation, they could contract in manifold other ways, but they are drawn into a selective, superordinate dynamics. Thus, the organizing cause of the muscle actions is my speaking (*downward*), which in turn is realized by a complex but constrained dynamics of physiological mechanisms (*upward*).

However, the same applies to the neuronal activity in motor and other areas of my brain: there is no place where an efficient-causal chain of “speech events” would begin. Rather, the neuronal processes proceed in this precise way because *I am speaking* these words, consciously spanning the intentional arc of the sentence over time, and roughly anticipating the meaning of the sentence and the next words to come. In other words, my embodied intentions and protentions are able to organize their physical implementation with the potential *to even achieve a future state that does not yet exist*. On a more basic level, such temporal loops also enable the *allostasis* mentioned above, by which conscious organisms regulate their needs in advance (Sterling, 2012). The coupling of an organism’s protentions and the corresponding environmental affordances act as a higher-order cause of the respective interaction. As overarching and future-directed enactments of life, conscious processes may thus be effective in the behavior of a living being without “acting on brain processes” in an external way.

In order to avoid any connotation of such efficient cause, one could also speak of an “implicational causality” (de Haan, 2020, p. 119): *by way of* thinking or speaking, I – as a living being – also realize certain organized processes in which ordered activities of neurons and muscles are implied; this happens inadvertently, as it were, similar to water molecules being drawn into a whirlpool that nevertheless consists of them. The whirlpool as form or order *implies* their specific movements without acting upon them. Thus, the complete cause of my speaking is neither my tongue nor my brain, but *I am this cause myself as a living being*. In each conscious action – walking, speaking, writing, or thinking – the living being as a whole acts as the forming, selecting, and organizing cause.

Again, circular causality does not mean external causation nor an interaction of mind and body but a relation of implication or global-to-local encompassing. Let us take the example of anxiety once more. A threatening situation, for example, an imminent loss of my job, induces growing anxiety, and this anxiety is obviously motivated by my former experiences and my subjective view of the current situation. On the other hand, changing from the personalistic to naturalistic stance, a neuroscientist might examine my brain in an fMRI scanner, zooming in, so to speak (de Haan, 2020), and find an increased activity in my amygdala. This activity is not the *cause* of my anxiety, however. The neuroscientist only turns to the physical aspect, with a narrow focus on the specific brain activity involved, leaving aside the circular interaction of the brain, body, and environment. Only the wider view, namely, considering the aspect of embodied subjectivity, its situatedness, and its history, provides a full explanation of my anxiety. On the other hand, it is not my anxiety that *causes* my amygdala to get activated – at least not in the usual sense of causality where cause and effect may be separated, one following the other. Much more is it that embodied subjectivity *constrains* or orders the patterns of brain activity involved.

Hence, there is no external causal relation between the experiential and neurophysiological aspects, because each refers to one and the same life process, looked at with a wider or a narrower focus. When I am anxious, there is no causal impact from either my brain activity to my experience or the other way around: rather, my having this experience *implies* certain brain activities, by way of circular causality or implication. Brain processes certainly enable my experience (upward causation), but the experiential aspect is wider with regard to both space and time. Only my relation to the current situation as a whole and my history of interactions with similar situations can explain my anxiety and the neural processes connected to it (downward causation). And only my anxiety as a future-directed subjective experience is able to motivate and organize the physical actions required for avoiding the threats I anticipate. Hence, via circular causation, embodied subjectivity as the integral of the brain–body–environment system is actually effective in the world, for it encompasses the physical processes necessary for its effect.

### Diachronic Circularity of Process and Structure

The impact of embodied subjectivity on the course and formation of physical processes and structures becomes even more obvious if we turn to the diachronic aspect, that is, the *development* of the individual human being. This may be described as a continuous *incorporation* of lived experience, in the sense suggested by Merleau-Ponty (1962, p. 192): “The body is solidified or generalized existence, and existence a perpetual incarnation.” In other words, existence as lived experience leaves its traces in the structure of the body, in particular in its neural structures. Development, learning, and memory formation may thus be conceived as a circularity of living *process* and solidified *structure*, continuously modifying each other. I will describe this diachronic circularity in more detail.

As research into neuroplasticity has amply shown, each bodily experience or behavior induces changes in the highly plastic matrix of the brain, mediated by epigenetic alterations of cellular functions and resulting in more adaptive dispositions and patterns of neural activity. This includes changes in the synaptic structure of neural networks, in the connectivity strength between brain regions, or even an anatomical enlargement of brain areas involved (McClung and Nestler, 2008; May, 2011). Thus, motor exercise, musical training, memorizing, meditation practice, and psychotherapy have been shown to durably change brain structure and activity (Goldapple et al., 2004; Draganski et al., 2006; Vestergaard-Poulsen et al., 2009; Dayan and Cohen, 2011; Ker and Nelson, 2019). In all these cases, the incorporation of experience in the form of altered neural dispositions results in an ever smoother performance, in acquired skills or habits.

Importantly, *conscious attention* obviously plays a crucial role for these top-down structuring effects. This was shown, among others, in a study by Recanzone et al. (1993) who trained monkeys to pay discriminative attention to either sound or touch stimuli presented to them simultaneously. After 6 weeks of the trial, a differential result emerged: in the monkeys attending to the sounds, the auditory brain area expanded, whereas the somatosensory area increased in monkeys attending to touch (for a similar experiment on the effect of discriminative attention in rats, see Polley et al., 2006). Conscious experience and attention thus act as “order parameters,” differentially constraining the current patterns of neural activation and thus also determining the long-term structuring of brain networks.

The extent to which the mammalian brain is already formed by interactive experience during early ontogeny has been impressively demonstrated by Mringanka Sur and his research team who induced a far-reaching cortical reorganization in newborn ferrets (Melchner et al., 2000; Sur and Rubenstein, 2005). They severed one of the ferrets’ optic nerves, so that the stump grew together with the part of the thalamus that usually transmits impulses from the auditory nerve to the auditory cortex. Now, visual stimuli, in dependence on the ferret’s motor activity, reached a brain region that usually processes acoustic signals. Surprisingly, the brain adapted to the sensorimotor patterns produced by the organism–environment interaction: in the course of several weeks, the auditory cortex became a visual cortex. It even developed orientation-selective cells that are characteristic of the visual cortex, so that the ferrets were finally capable of seeing with the eye concerned.

As it turns out, it ultimately depends on the sensorimotor interaction and its specific patterns of neural excitation, which task a cortex region ultimately takes on. Similar cortical reorganizations can also be observed in humans after brain lesions or strokes where patients can re-learn major skills by continuous exercise and training; language and orientation functions can even be taken over by the other hemisphere (Dimyan and Cohen, 2011). All this may be expressed by the principle “form follows function”: *consciously interacting with the environment induces the development of the neuronal structures necessary for ever smoother interaction and experience*.

**FIGURE 2:**
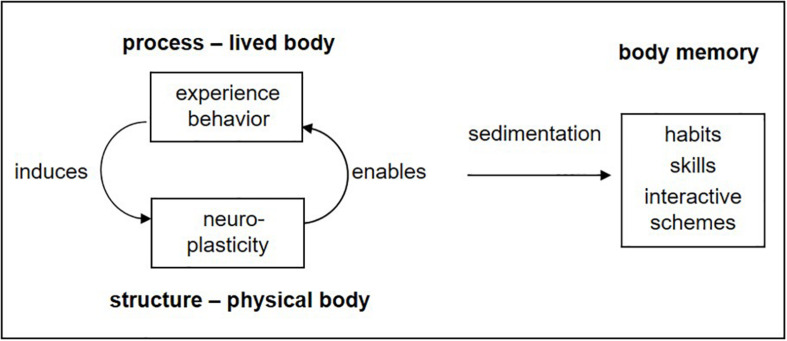
Circularity of process and structure: learning as transformation of experience or behavior into organic (in particular neural) dispositions (adapted from Fuchs, 2018, p. 140).

This is the basis of learning, memory, and development from birth on: a downward effect of the superordinate body–environment system, corresponding to the subjective experience, induces adaptive changes in the neural substrate, which in turn enable improved functioning (Figure 2). It may also be described as a continuous circularity between *experiential process and organic structure*, or in other words, between *lived body and physical body.* Over time, repeated experiences are sedimented or incorporated in what may be termed *body memory* (Fuchs, 2012b, 2018), namely, the totality of dispositions, habits, skills, and interactive schemes acquired by an individual in the course of his or her development.

Of course, there are no two separate processes going on, one experiential and one physiological, which would somehow act on each other. Rather, we are looking at two aspects of one and the same process: the one implying the lived interaction within the wider system of organism and environment and the other having a narrower focus on the physiological processes and the continuous reorganization within the brain, which turns process into structure. Hence, there is circular causality, downward influencing, and upward enabling but no causal interaction between the aspects.

Switching between both aspects in the diachronic sequence, we can also speak of a *spiral-shaped development:* lived body and organic body, each considered as aspects of the life process, mutually influence and modify each other. As superordinate processes, the lived body’s interactive experiences become organic dispositions, which in turn enable new forms of experience. The dialectics of *Leib* and *Körper* unfold in time and become the dynamics of lived (present) and sedimented (past) experience, or of process and structure mutually turning into each other – which is precisely what we call *learning and development*.

**FIGURE 3:**
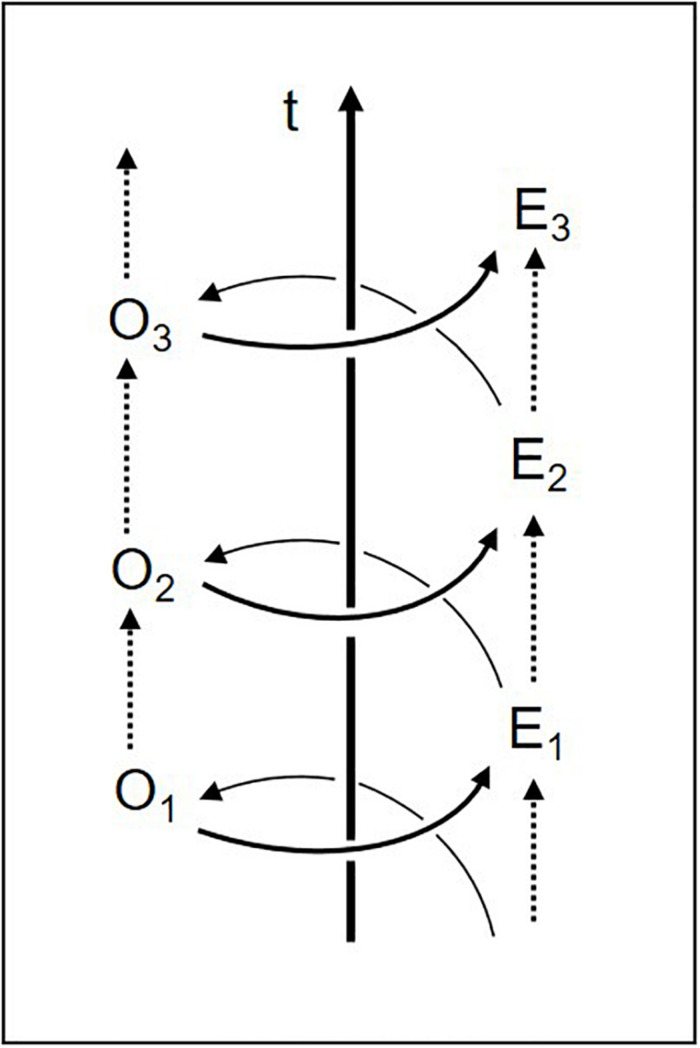
Co-evolution of organism (O) and environment (E) over time (t) (adapted from Fuchs, 2018, p. 103).

In the diachronic dimension, then, the two-dimensional circle of body–environment interaction actually becomes a *three-dimensional spiral* (it only appears as a circle when viewed from above, neglecting its diachronic axis; cf. Figure 3). Experience turns into the organism’s altered dispositions (O_1_, O_2_, O_3_, …), which change the perceived environment and its selected affordances (E_1_, E_2_, E_3_, …), thus in turn enabling new experiences, and so on. Perceived affordances are thus shaped by the history of the structural coupling of organism and environment^10^. In early childhood, for example, objects take on special relevance once infants acquire certain manipulatory skills. As Eleanor Gibson has shown, sensorimotor learning is based on the infant’s exploratory activity and environmental feedback, leading to a continual increase in perceiving what is doable (Gibson, 1991, 2000). Every acquisition of new motor skills – reaching, walking, swimming, driving, sewing, and handwriting – produces new affordances throughout life (Adolph and Kretch, 2015).

This is obviously not a merely individual development – most capacities, customs, and cultural techniques are acquired in the course of *embodied social practices* such as imitation, joint attention, and cooperative learning. The social and cultural environment with its shared practices becomes the decisive “ontogenetic niche” for scaffolding the infant’s development and selecting appropriate neural structures (Tomasello, 1999; Kendal, 2011). The embodied mind is thus intersubjectively formed from birth on. To give one example, infants have a universal potentiality for speech and articulation, which through acquiring the mother tongue is gradually restricted to a culture-bound pattern. Therefore, in the first months of life, babies can still distinguish more phonemes than the adults of their culture (Markowitsch and Welzer, 2009, p. 160–164). Via implicational or downward causality, the plastic matrix of their brains is shaped by the higher-order patterns of social interactions (Kuhl, 2010). These interactions restrict and determine what now appears to the baby as meaningful social affordance, namely, familiar verbal sounds, whereas foreign sounds remain meaningless. Of course, the latter may still serve as affordances, yet only for babies from another culture. This is just one example of the spiral of process and structure that characterizes childhood development as a whole and continues later on – as the constant incorporation of experience or “perpetual incarnation of existence” (Merleau-Ponty, 1962). The cultural environment serves as a higher level system that scaffolds, selects, and constrains the formation of individual brain functions and corresponding capabilities.

A similar “spirality” can also be found in the *phylogenetic* development of *homo sapiens*: human culture gradually formed a new ecological niche, which acted as a superordinate formative field that favored and selected appropriate organic structures, including the higher structures of the human brain (Sterelny, 2010; Sutton, 2015). Another example is the evolution of the human larynx, which adapted to the cultural development of language: compared with other primates, it descended to a lower position, thus opening a unique resonance space for the differentiation of vowels and allowing the human tongue to move more freely, to the advantage of our phonetic repertoire (Fitch, 2000). Even though the crossover of the respiratory and digestive tracts resulting from the lowered larynx is dysfunctional in another respect (it may lead to choking and lethal aspiration), the further development of language obviously outweighed the disadvantage. Thus, in human evolution, we find again an analogous relation of *process and structure*: on the one hand, intercorporeality and interaction increasingly developed toward symbolic communication; on the other hand, these social processes shaped the structure of the human organism, although of course within an evolutionary, phylogenetic time frame.

This results in a *spiral of cultural and biological evolution*, and in the inherent connection of embodiment, social interaction, and culture (Durt et al., 2017). Humans create their own specific environment, consisting not only of material products of culture such as tools or artifacts but also of shared ways of sense-making and interaction that are established as symbols, codes, rituals, and habits. This constitutes a universe of novel, *cultural affordances*, which impregnate and structure the individual ontogeny. The “material culture” (Malafouris, 2013) and the symbolic culture have to be appropriated and incorporated by every new generation; this is crucially mediated and enabled by the “encultured brain” (Lende and Downey, 2012), which adapts to the cultural scaffolding on the basis of circular causality.

### Self-Formation: Modifying the Spiral

The processes of circularity and development mentioned so far were mostly involuntary; learning, habit, and skill formation were considered as part of the overall process of enculturation. However, it is characteristic of the human species that its members increasingly take ownership and responsibility for this development themselves. By their decisions and actions, by choosing a certain way of life and environment, individuals shape their own development, because the chosen way of life and environment feed back on their own becoming. Humans not only live their lives but also *lead* them, and through this, they form and cultivate themselves. This means that the spiral of process and structure is *deliberately* modified and directed to an anticipated goal.

There are two presuppositions for this individual self-determination:

a) *Relationship to oneself:* Based on the capacity of self-reflection, the individual is in a position to take a stance toward his or her own development. He or she is no longer determined by the higher-order system of cultural socialization but can detach himself or herself from the current situation and anticipate and evaluate possible alternatives of life.
b) *Embodied freedom*: As shown above, in each conscious action, the living being as a whole acts as the forming, selecting, and organizing cause – in accordance with the principle of downward causation. In humans, this principle is raised to a higher potential by the possibility of autonomous decision making. Free will should not be regarded as a purely mental feat, however; making a choice and acting according to it are rather the result of an “embodied freedom,” which integrates the entire bodily, affective, and cognitive situation of the person in each decision and its execution (see Fuchs, 2018, p. 236–243, for further explanation).

Objections to a concept of genuine human freedom are mainly rooted in latent dualistic intuitions, assuming this kind of freedom to rest on an immaterial mind steering the activities of neurons. By contrast, the concept of embodied freedom is based on circular or implicational causality; it regards decisions as superordinate, intentionally directed enactments of life performed by an embodied person – enactments that are enabled but not determined by the neuronal processes involved. Of course, the problem of free will cannot be discussed here in detail (cf. Banks et al., 2006; Gallagher, 2006; Murphy et al., 2009); suffice it to emphasize the fundamental change brought about by human freedom in the top-down processes of enculturation. All a person’s experiences and actions leave behind traces in the organism and thus change his or her dispositions, skills, and potentialities. A person’s being is continually becoming, but *this becoming is increasingly his or her own doing*. Through their decisions and actions, human persons shape their own development.

This new level of freedom creates a particularly human spirality, which we find already expressed in Aristotle’s concept of *hexis*, that is, a personal habitus and character that is continuously shaped through self-forming actions:

“The virtues we get by first exercising them, as also happens in the case of the arts as well. For *the things we have to learn before we can do them, we learn by doing them*, e.g., men become builders by building and lyreplayers by playing the lyre; so too we become just by doing just acts, temperate by doing temperate acts, brave by doing brave acts. […] This is why the activities we exhibit must be of a certain kind; it is because the states of character correspond to the differences between these. It makes no small difference, then, whether we form habits [*hexis*] of one kind or of another from our very youth” (Aristotle, 1925; my italics).

The italicized passage describes precisely the spiral of human learning, namely, shaping the body’s dispositions, skills, and habits through one’s actions, which are in turn increasingly enabled by these dispositions. This circularity extends to the sphere of moral actions: in the course of mental development, they become more and more self-determined, and through repetition and habitualization, they form a “virtuous” character. One might conclude that embodied subjectivity most clearly proves its effectiveness or its non-epiphenomenal character *when it directs its actions on itself* and thus produces a lasting self-forming and self-changing effect. This may be considered the highest stage of the principle of circularity.

## Conclusion

In this paper, I have studied the interrelation of lived or subject body (*Leib*) on the one hand and living or object body (*Körper*) on the other. Both were considered as complementary, irreducible, mutually constituting, and also mutually concealing aspects of the living being. They correspond to two different attitudes that we may adopt: in the personalistic attitude, we experience our own lived body from a first-person perspective or the other’s lived body from a second-person perspective. In the naturalistic attitude, we observe or investigate the physical body from a third-person perspective. Whereas the personalistic attitude and its corresponding aspect require a holistic view of the living being or the person, the naturalistic attitude allows for focusing on increasingly narrow sections and details of the physical body, albeit at the price of losing the phenomena of life and mind. A person, that is, a living, embodied subject, can only be perceived as such by another embodied subject in the personalistic attitude.

In order to further investigate the relation of both aspects and the “body–body problem,” I have interpreted the intertwinement of subject body and object body on the basis of the concept of *circularity*. First, embodiment shows a circular structure, because it is based (a) on the cycles of homeostatic self-regulation between the brain and body and (b) on the sensorimotor cycles between the brain, body, and environment. The first cycle is the foundation of the feeling of being alive, or the pre-reflective background feeling of the body itself. The second cycle is the basis of bodily “being toward the world” (Merleau-Ponty), or of situated, enactive subjectivity. Here, in terms of ecological psychology, living beings and their surroundings constitute an interactive system, with each constituent being reciprocal to the other: what we perceive are not objects as such but objects to deal with, or the functional relations between self and world. In other words, there is a mutual interdependence of the bodily dispositions of sense-making and the affordances of the environment disclosed by these dispositions.

In both kinds of cycles, the ongoing circularity of the processes involved does not allow for an internalistic account on the basis of representations in the brain, which could in principle be separated from their source. There is no component within the cycles that represents another component, in the sense that it could stand for it while it is absent; “inside” and “outside” are functionally coupled and may not be separated. Hence, bodily self-awareness as well as conscious being-in-the-world can no longer be localized in the brain; instead, they are the *integral manifestation* of the brain–body–environment system, or of the overarching process of life encompassing the whole organism. This conception unites the first-person phenomenology of the lived body with a systemic approach provided by both enactivism and ecological psychology.

In order to establish the effectiveness of embodied subjectivity, I have further used the concept of *circular causality*, which characterizes the relation of parts and whole within the living organism as well as within the organism–environment system. Downward causation enables an account of embodied subjectivity as being equivalent to an ordering or forming cause of a living being’s actions, while avoiding dualistic assumptions of the “mind acting on the body.” It is a causation by global-to-local implication, not a separate mental activity or impact. Importantly, this kind of causation includes the possibility of achieving future states anticipated by embodied intentions and protentions. Hence, only the wider view of the subject as embodied and situated, with both regard to space and time, is able to fully explain a person’s experience and behavior.

As a next step, I have described the interrelation of lived and physical body as a *circularity of experiential process and (neuro-)physiological structure* underlying development and learning. Here, the circular causality of higher- and lower-level processes is considered as unfolding in the *diachronic* dimension, based on the plasticity of the brain. Subjective and intersubjective experience constitutes a process of sense-making that includes cerebral processes so as to form modified neuronal structures, which in turn enable altered future interactions. Only conscious experience contains the intentional and meaningful relations to the environment whose correlates are functionally and morphologically inscribed in the brain throughout the course of socialization. This results in a *spiral-shaped development:* lived body and organic body mutually influence and modify each other. This is not only an individual development, however; the social and cultural environment with its shared meanings, habits, and artifacts constitutes the crucial *ontogenetic niche* for the individual formation of the brain. Analogously, human culture has also provided the decisive scaffolding for the *phylogenetic evolution* of the organic (in particular, neural) structures of the human being.

A final step is reached with the possibility of *shaping one’s own development*, which arises with the relation to oneself and the autonomy of the person. This is the circularity of freedom: by choosing one’s actions and way of life, one also shapes the body’s dispositions, skills, and habits which increasingly favor those actions. Individuals are not just the result of the organic, social, and cultural conditions, which have contributed to their development; instead, they take control and responsibility of their own becoming by choosing the experiences, actions, and situations that feed back on their development. This self-determination is based on circular causality as a presupposition of free decisions and actions, and on the human capacity for taking a stance toward one’s own being and becoming.

In conclusion, the proposed – yet certainly not exhaustive – solution to the body–body problem may be summarized as follows:

a) Lived body and living body correspond to two complementary, irreducible, but intertwined *aspects of the living being*, regarded from two different perspectives or attitudes.
b) The *living body as a whole* is the constitutive basis of the subjective lived body; or in other words, the latter is equivalent to the integral experience that we have as living organisms in relation to our (physical, social, and cultural) environment. The brain is not the locus of subjectivity but only a mediating component of the cycles of self-regulation, sensorimotor, and social interaction, in which the life of a human person consists.
c) The lived body or embodied subjectivity has a top-down, ordering, and constraining effect on the physical body and its processes, and over time, a formative effect on its (neuro-)physiological structures. These effects are mediated by *circular causation* or by way of *implication*.

d) Accordingly, lived body and living body, *Leib* and *Körper*, mutually enable and constitute each other. This is what defines our embodiment as human persons.

## Author Contributions

The author confirms being the sole contributor of this work and has approved it for publication.

## Conflict of Interest

The authors declare that the research was conducted in the absence of any commercial or financial relationships that could be construed as a potential conflict of interest.

## Footnotes

1 Consciousness shows itself to others only through the expressivity of the lived body (be it in emotional or verbal expression), that means, in the personalistic attitude. The same applies to life, however. One could object that the life sciences also deal with life from a third-person perspective. This is true, but when speaking of living beings, they already presuppose our coexistence or “conviviality” with life, which lets us grasp living entities in accordance with our own self-experience, namely, as moving themselves, sensing, striving, and “being up to something.” Inasmuch as the life sciences abstract, in a second step, from our self-experience of life, they conceive of living beings merely as mechanisms or machines – and thus, no longer as living or animate. “Life can be known only by life” (Jonas, 2001, p. 91); or in other words, life cannot be fully grasped from a third-person perspective.

2 A representational relation in the traditional sense implies that an internal state of a system (usually the brain) “stands for” an external state of affairs. According to Piccinini (2018), this includes four elements: (i) a homomorphism between a system of internal states and their target, (ii) a causal connection from the target to the internal states, (iii) the possibility for the internal states to be decoupled from their target, and (iv) a role in action control. In other words, the computational process realizing the representation is causally connected to but in principle separable from the peripheral body or from the environment (see also Markman and Dietrich, 2000).

3 On the impossibility of a “brain in a vat” modeling the world without constitutive embodiment, see also Cosmelli and Thompson (2011).

4 In a similar vein, Gibson has pointed to the anchoring of perception in self-awareness: “This is only to reemphasize that exteroception is accompanied by proprioception – that to perceive the world is to coperceive oneself” (Gibson, 1979, p. 141). However, I prefer the term interoception here, because the basic sense of self or self-affection is derived from the deep body (visceral feedback to the brain) rather than from the proprioception of “legs, hands, and mouth” (Gibson, 1979).

5 These are feedforward mechanisms that inform the sensory system of imminent self-movements (Holst and Mittelstaedt, 1950). In this way, for example, the movements of the eyes are taken into account by the sensory system, because otherwise, the perceived surroundings would start to sway with every eye movement. Circularity is thus found already on the subpersonal level.

6 The currently fashionable concepts of the brain as a “prediction machine” (see, e.g., Clark, 2013; Hohwy, 2013) restrict and reduce the potentiality of the whole organism–environment system to an internal computing mechanism. However, the anticipatory structure of the perception–action cycle is crucially based on the movable body as well as on the affordance profile of the environment; the brain only connects and mediates these properties and potentialities of the system. For an enactive critique of the “predictive coding” concept, see also Gallagher (2017, pp. 15–20).

7 On this, see Fuchs (2018, pp. 145–155) and Kevin and Shaun (2020, this issue). Gibson also speaks of the sensory system *resonating* with global changes in the perceptual field: “In the case of the persisting thing, I suggest, the perceptual system simply extracts the invariants from the flowing array; it resonates to the invariant structure or is attuned to it” (Gibson, 1979, p. 249).

8 In algebra, the integral enables the calculation of an area that is bounded by a function over a certain basis. I use it as a metaphor to signify the integration that consciousness achieves over an extended basis, without being separable from that basis as a system of “representations.”

9 It is also through the functional sensorimotor cycles that the object body is constituted in experience. Whereas the basal feeling of being alive corresponds to the internal, deep body or “body-as-subject,” that is, the endogenous source of experience that cannot itself become an object, the body re-appears on the level of directed, sensorimotor relations to the environment, namely, as an *object* of proprioceptive, tactile, and visual perception, or as “body-as-object” (a special object though, as it remains always present). Hence, the internal body conveys the background state of being-directed-toward something – the body as medium – whereas the external body is the body that we become conscious of, or that we can use like an instrument. Whereas the body-as-subject is primarily constituted on the level of subcortical brain structures coupled with the visceral body, the body-as-object requires cortical structures connected with the sensorimotor body (cf. also Solms, 2013; Fuchs, 2018, p. 117f.).

10 In enactive terms: “What constitutes the world of a given organism is enacted by this organism’s history of structural coupling” (Varela et al., 1991, p. 202).
